# Serological and Progression Differences of Joint Destruction in the Wrist and the Feet in Rheumatoid Arthritis - A Cross-Sectional Cohort Study

**DOI:** 10.1371/journal.pone.0136611

**Published:** 2015-08-28

**Authors:** Yosuke Hamamoto, Hiromu Ito, Moritoshi Furu, Motomu Hashimoto, Takao Fujii, Masahiro Ishikawa, Noriyuki Yamakawa, Chikashi Terao, Masayuki Azukizawa, Takahiro Iwata, Tsuneyo Mimori, Shuichi Matsuda

**Affiliations:** 1 Department of Orthopedic Surgery, Kyoto University Graduate School of Medicine, Kyoto, Japan; 2 Department of the Control for Rheumatic Diseases, Kyoto University Graduate School of Medicine, Kyoto, Japan; 3 Department of Rheumatology and Clinical Immunology, Kyoto University Graduate School of Medicine, Kyoto, Japan; 4 Center for Genomic Medicine, Kyoto University Graduate School of Medicine, Kyoto, Japan; Northwestern University Feinberg School of Medicine, UNITED STATES

## Abstract

**Objective:**

To investigate clinical and radiological differences between joint destruction in the wrist and the feet in patients with RA.

**Methods:**

A cross-sectional clinical study was conducted in an RA cohort at a single institution. Clinical data included age, sex and duration of disease. Laboratory data included sero-positivity for anti-cyclic citrullinated peptide (CCP) antibody and RF. Radiological measurements included Larsen grades and the modified Sharp/van der Heijde method (SHS) for the hands/wrists and the feet. Statistical analyses were performed using the Kruskal—Wallis H-test, a dummy variable linear regression model and multivariate logistic regression analysis with 95% confidence interval and odds ratios.

**Results:**

A total of 405 patients were enrolled, and 314 patients were analysed in this study. The duration of disease in the foot-dominant group was significantly less than that in the wrist-dominant group. When patients were subdivided by duration of disease, the Larsen grade of the feet was significantly higher than that of the wrist in the first quadrant subgroup, but this was reversed with increasing duration of disease. Anti-CCP status was a significant predictive factor for joint destruction in the wrist but not in the feet, while RF status was not predictive in either the wrist or the feet.

**Conclusions:**

Joint destruction in the feet started earlier than in the wrist, but the latter progresses faster with increasing duration of disease. Anti-CCP status predicts joint destruction in the wrist better than in the feet.

## Introduction

RA is characterized as a disease that causes long-standing, accelerating functional impairment because of progressive joint destruction throughout the body. It has been considered that joint inflammation affects daily function in the early stages of the disease mainly because of pain and swelling of the affected joints, while joint destruction and deformity significantly worsen the functional impairment in the established stages of the disease. Many recent reports show that joint destruction starts in the very early stages of RA and is much more rapidly progressive than in the later stages [1, 2], which may prompt rheumatologists to adopt more intensive treatment strategies from the time of diagnosis. Some reports even show that joint destruction has already started by the clinical onset of RA, and emphasize the importance of aggressive treatment at the very beginning of the disease [3–6]. However, the processes by which joint destruction starts and progresses remain largely unknown, especially from a perspective of which joints are affected.

The wrist joint is one of the most frequently affected joints in RA, and has significant diagnostic and therapeutic value as a small joint in the revised classification and remission criteria [7, 8], even though it seems to be a ‘large joint’ from an anatomical point of view. Moreover, the importance of the wrist in daily function weighs much more heavily than that of other small joints such as metacarpo-phalangeal (MCP) or proximal interphalangeal (PIP) joints [9] because of its size and its regional location in the upper extremity. In contrast, the metatarso-phalangeal (MTP) joints, also among the most frequently affected types of joint, attract much less attention, exemplified by the fact that most clinical disease activity scores do not include these joints. However, a series of previous reports have shown their critical influence in daily living for patients with RA [9–12]. Moreover, tight control of RA disease in fact increases the frequency of orthopaedic reconstructive surgeries of the feet, even though the majority of these patients are in remission or have low disease activity [13], and the importance of the feet has been recently revised by rheumatologists and patients with RA. Taken together, joint destruction both in the wrists and the feet undoubtedly affects daily function in the long term, but comparison of these two sites in terms of progression of destruction largely remains to be investigated. If differences in progression and independent risk factors are established, it may be possible to adjust therapeutic strategies based on this knowledge.

A number of attempts have been made to predict the progression of RA using disease activity, joint destruction at the time of diagnosis, and laboratory biomarkers. Serological biomarkers have been recognized as crucial factors not only for diagnosis of the disease but also for predicting to some extent the severity of disease. Historically, RF has been the marker on which rheumatologists have relied heavily, but the presence of anti-CCP antibody has recently attracted much more attention because of its reliability as a predictor of disease course [2, 14–17]. However, the differences between these two crucial factors remain largely undifferentiated, especially in relation to joint destruction.

Considering the crucial effects of joint destruction in RA, the mechanism by which joint destruction starts and progresses and which factors are independent risk factors should be identified in a clinical study. Therefore, we conducted a cross-sectional diagnostic study in an RA cohort to investigate the progression of joint destruction and to identify independent risk factors. We specifically asked the following questions: 1) is there any difference between the wrist and the feet in terms of the start and/or the progression of joint destruction, and 2) are there any independent risk factors to predict joint destruction of the wrist and/or the feet, respectively?

## Methods

### Study Population

Consecutive patients from the Kyoto University Rheumatoid Arthritis Management Alliance (KURAMA) cohort were enrolled between April and December 2012 for a cross-sectional diagnostic study. The KURAMA cohort was established in May 2011 at the Center for Rheumatic Diseases at Kyoto University Hospital with the aim of tightly controlling RA, and the participants’ clinical and laboratory data were prospectively utilized for clinical investigations as reported previously [18–21]. Each year, 400 to 600 patients are consecutively registered in the KURAMA cohort.

The inclusion criteria for this study were as follows: patients with written informed consent, over 18 years of age, having a complete set of data, and fulfilling the American College of Rheumatology/European League against Rheumatism classification criteria for RA from 1987 or 2010. Exclusion criteria were patients who refused enrolment in the study, for whom any data were missing, or who had undergone any surgery on either wrist or any MTP joint. The study was designed in accordance with the Declaration of Helsinki and was approved by the Medical Ethics Committee of Kyoto University Graduate School and Faculty of Medicine before the start of the study.

### Data Collection

#### Clinical assessments

Clinical data included age, sex, duration of disease, swollen joint counts, tender joint counts and patient’s and physician’s global assessment of disease activity. Laboratory data included C-reactive protein (CRP), erythrocyte sedimentation rate (ESR), and levels of RF, anti-cyclic citrullinated peptide antibody (anti-CCP antibody), and matrix metalloproteinase-3 (MMP-3). We evaluated disease activities using the Disease Activity Score 28-ESR (DAS28-ESR) and the Simple Disease Activity Index (SDAI). The use and doses of methotrexate, glucocorticoids and biological disease-modifying anti-rheumatic drugs (bDMARDs) were also recorded. All clinical and laboratory data and radiographs were collected at the same time point.

#### Radiographic measurements

Standardized radiographs of the wrists/hands and the feet (postero-anterior view) were obtained according to the modified Sharp/van der Heijde method (SHS) [22, 23]. We decided to analyze the MTP joints that are among the most frequently affected joints for which there is an established grading system in the Larsen grade, compared with other joints in the feet. To compare the MTP joints, we selected the right wrist joint, because the wrist is one of the most frequently affected and most influential joints for daily function in patients with RA [9]. We did not use the MCP joints that would be comparative joints to the MTP joints but has shared significance with PIP joints in RA from a pathophysiological point of view. The radiographs were analysed by two experienced rheumatologists (MF, NY) using the Larsen grade [24] and SHS. The readers were blinded to information about the subjects. The reported results were the average of the two grades or the two scores reported by the two readers. The cut-off point for established joint destruction was defined as the grade including one-third of the patients who had the most severe joint destruction evaluated by Larsen grade. We decided to define established joint destruction in the right wrist joint as representing the joint destruction of both wrist joints and a certain number of MTP joint with a certain Larsen grade from the both feet as representing that of the feet. The cut-off point resulted in using Larsen grade 3 or more in the wrist (37.3% of the total study population), and Larsen grade 3 or more in at least two of the MTP joints in the feet (36.6% of the total study population). When SHS was used, the joint destruction was defined as greater than the median SHS for the entire population both in the wrist/hand and in the feet. The cut-off point for the wrist/hand was 56, and for the feet was 16.6. The total study population was subdivided into four subgroups according to the Larsen grades in the wrists and the feet: a Wrist—Foot group that had established joint destruction both in the right wrist and in the feet (n = 62); a Wrist group that had established joint destruction only in the wrist (n = 74); a Foot group that had established joint destruction only in the feet (n = 53); and a None group that did not have established joint destruction in either the wrist or the feet (n = 125). We also subdivided the entire population into four quadrant subgroups by duration of disease (less than 4, 4–13, more than 13–22, and more than 22 years).

### Statistical Analysis

The JMP Pro statistical package, version 11.0.0 (SAS, Institute Inc., Cary, NC, USA) was used for all the statistical analyses, and a *P* value less than 0.05 was considered significant. Data were expressed as average and standard deviation (SD) for continuous variables and numbers and percentages for categorical variables unless otherwise stated. When a trend over the duration of the disease was evaluated, a dummy variable linear regression model was used to analyse the four quadrant subgroups. Statistical analyses were performed using the Kruskal—Wallis H-test for comparison of four groups, and regression analyses. Factors affecting joint destruction in the wrist or the feet were analysed using correlation analysis and multivariate logistic regression analysis with 95% confidence interval (95% CI) and odds ratio (OR).

## Results

**Fig 1:**
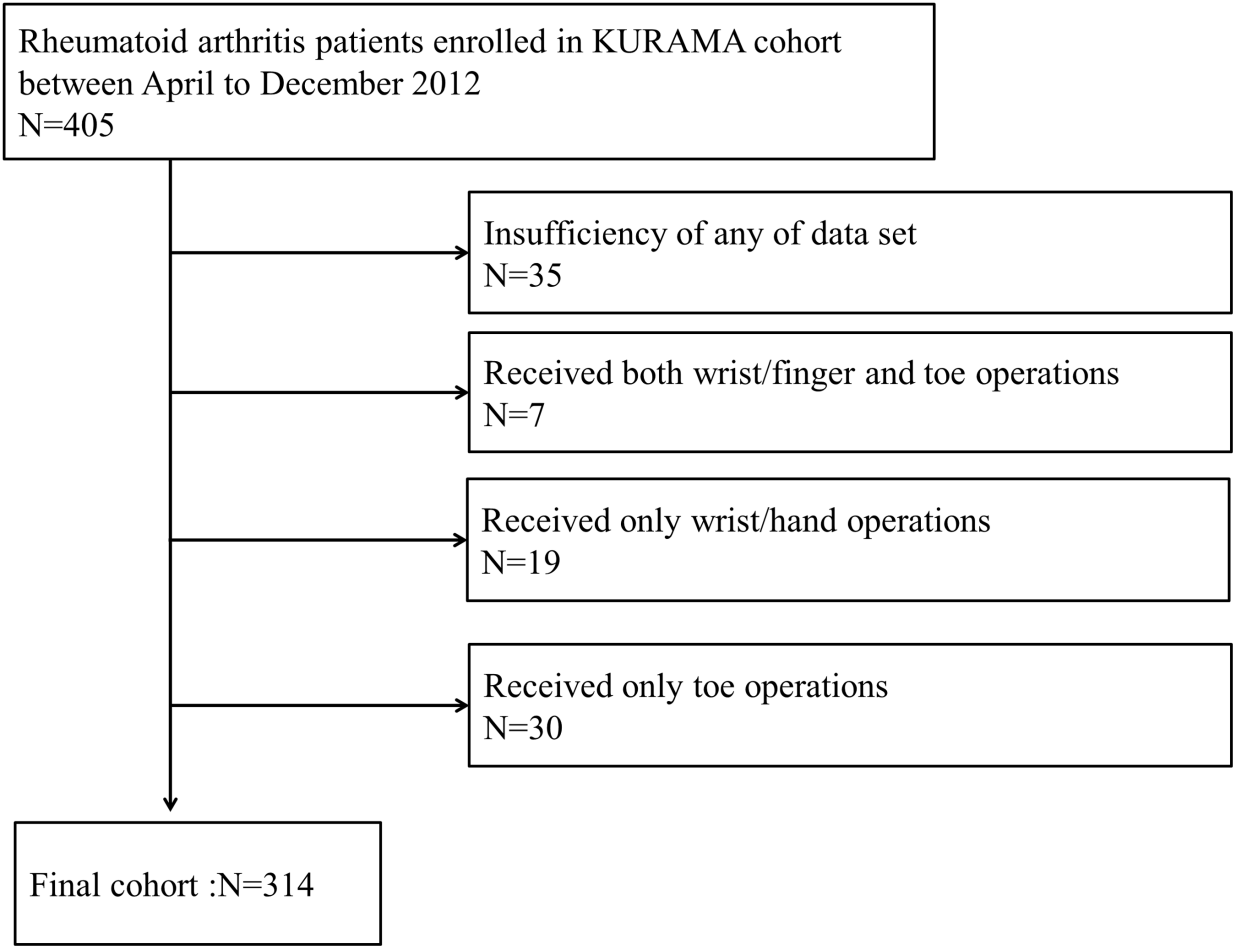
Flow chart of the study.

A total of 405 patients were enrolled during the study period, of which 56 had undergone previous surgery on either the wrist or one of the MTP joints and were included only in the analyses mentioned. Thirty-five patients lacked some of the data set, meaning that 314 patients (77.5%) were analysed in this study (Fig 1). We found no significant differences in the average age, the proportion of females, the average duration of the disease, positivity of anti-CCP antibody or RF between included and excluded patients. The average age at enrolment was 62.4 (23–92) years, and the average duration of disease was 13.0 (0.1–64) years (Table 1). Rates of positivity for anti-CCP antibody and RF were 81% and 80%, respectively. More than 38% of patients who did not have established joint destruction in the feet had established joint destruction in the right wrist, while more than 18% of the patients who did not have established joint destruction in the right wrist had established joint destruction in the feet.

**Table 1:** Demographic data.

	Average(min-max)or n (%)
Age at survey (year)	62.4 (23–92)
Disease duration (year)	13.0 (0.1–64)
Steinbrocker Stage	I; 58,II; 88, III; 59, IV; 109
DAS28-ESR	3.13 (0.084–6.59)
Anti-CCP positivity	254 (81%)
RF positivity	252 (80%)
CRP (mg/dl)	5.7 (0–65)
MMP-3 (ng/ml)	127.8 (19.9–655)
HAQ	0.751 (0–3)
bDMARDs	95 (30%)
Steroid, average amount (mg)	166 (53%), 4.8
MTX, average amount (mg)	7.2

*DAS28;* Disease Activity Score 28, *ESR;* erythrocyte sedimentation rate, *anti-CCP;* anti-cyclic citrullinated peptide antibody, *CRP;* C-reactive protein, *RF;* rheumatoid factor, *MMP-3;* matrix metalloprotenase-3, *HAQ;* health assessment questionnaire, *bDMARDs;* biological disease modifying anti-rheumatic drugs, *MTX;* methotrexate

**Fig 2:**
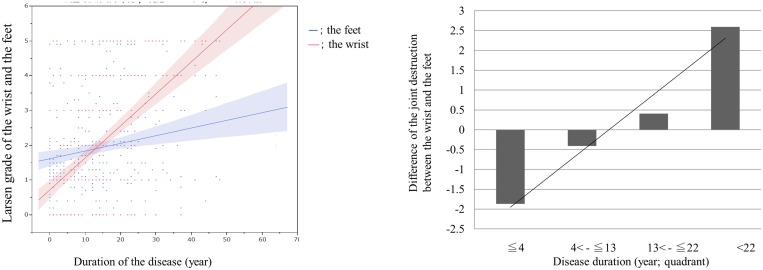
(A) Comparison of joint destruction of the wrist and the feet in the duration of the disease. Larsen grade of the feet was significantly higher than that of the wrist in the first subgroup (p<0.001). (B) Comparison of difference of the joint destruction between the wrist and the feet in Larsen grade. *P* < 0.001.

When patients were subdivided by duration of disease, the Larsen grade of the feet was significantly higher than that of the wrist in the first subgroup (less than 4 years duration, Fig 2A). A dummy variable linear regression analysis showed that the differences between the wrist and the feet became significantly greater with increasing duration of disease (*P* < 0.0001, Fig 2B), indicating that the progression of the Larsen grade in the wrist was faster than that in the feet (S1 Table). We replicated the same analyses of the hands and the feet using SHS and found that a similar significant trend existed (S1 Fig).

We compared the demographic data of the four subgroups (None, Foot, Wrist, and Wrist-Foot subgroups). Statistical analyses showed that the duration of the disease and the Health Assessment Questionnaire scores of the Foot and None groups were significantly less than those of the Wrist—Foot and Wrist groups, and the DAS28-ESR scores of the Foot and None groups were significantly less than that of the Wrist—Foot group (Table 2). The rate of anti-CCP and RF positivity in the None group was significantly less than in the other three groups.

**Table 2:** Comparison of 4 subgroups based on wrist/foot differences of joint destruction in Larsen grade.

		Wrist-Foot (n = 62)	Wrist (n = 74)	Foot (n = 53)	None (n = 125)	P value
age	(SD)	64.0 (10.6)	62.5 (11.4)	60. 1(13.5)	60.8 (14.2)	n.s.
sex	number (%)	57 (91.2)	63 (85.0)	48 (91)	157 (86)	n.s.
disease duration	mean (SD)	22 (9.54)	17.7 (11.2)	8.79 (10.6) * §	9.24 (8.60) * §	<0.01
DAS-ESR	mean (SD)	3.48 (1.19)	3.29 (1.14)	3.00 (1.18) *	3.04 (1.17) *	<0.05
HAQ	mean (SD)	1.17 (0.85)	0.84 (0.749)	0.67 (0.69) * §	0.64 (0.70) * §	<0.01
Anti-CCP positivity	number (%)	56 (90.3)	66 (89.1)	46 (86.8)	86 (68.8) * § †	<0.01
RF positivity	number (%)	55 (88.7)	62 (83.7)	43 (81.1)	92 (73.6) * § †	<0.01
bDMARDs	number (%)	21 (34)	21 (28)	13 (24)	50 (28)	n.s.
The amount of MTX	mean (SD)	4.47 (4.75)	6.03 (4.57)	5.28 (4.30)	4.91 (3.79)	n.s.
The amount of steroid	(SD)	4.36 (2.09)	5.26 (3.67)	5.31 (2.61)	4.64 (2.92)	n.s.

*DAS28;* Disease Activity Score 28, *ESR;* erythrocyte sedimentation rate, *HAQ;* health assessment questionnaire, *anti-CCP;* anti-cyclic citrulinated peptide antibody, *RF;* rheumatoid factor, *bDMARDs;* biological disease modifying anti-rheumatic drugs, *MTX;* methotrexate.

*: statistically significant against Wrist-Foot group (p<0.05),

^§^; statistically significant against Wrist group,

^†^; statistically significant against Foot group, n.s. denotes not significant.

We further investigated whether any background characteristics could predict the progression of joint destruction in the wrist or the feet. Multivariate logistic regression analysis found that anti-CCP status was a significant factor for predicting established joint destruction in the wrist (OR 4.19, 95% CI 1.63–11.92) but not in the feet (OR 0.73, 95% CI 0.36–1.45), while RF status was not significant in either the wrist or the feet (Table 3). Similarly, when we analysed joint destruction as defined by SHS separately in the wrists/hands and the feet, anti-CCP status was a significant factor for predicting joint destruction in the wrist (OR 4.48, 95% CI 1.83–11.97) and in the MTP joints (OR 2.41, 95% CI 1.12–5.44), while RF status was not predictive for either the wrist or the feet (S2 Table). These data indicate that anti-CCP status is strongly associated with joint destruction in the wrists, but less so with that in the feet. The use and the dose of medication (methotrexate, steroids, and bDMARDs) were not associated with the progression of joint destruction (Table 2), indicating that disease activity was well controlled and was not influencing the specificity of joint destruction in most patients.

**Table 3:** Prognostic factors of the joint destruction in Larsen grade and covariables (OR, 95%CI).

	Wrist	Feet
Duration of the disease	1.14 (1.10–1.17)*	1.03 (1.01–1.05)*
Anti-CCP positivity	4.19 (1.63–11.92)*	0.73 (0.36–1.45)
RF positivity	0.93 (0.39–2.27)	0.54 (0.26–1.08)

*OR;* odds ratio, *95%CI;* 95% confidence interval, *DAS28;* Disease Activity Score 28, *anti-CCP;* anti-cyclic citrulinated peptide antibody, *RF;* rheumatoid factor,

*: p<0.05

## Discussion

This cross-sectional cohort study attempted to analyse the differences in joint destruction in the wrists and the feet of patients with RA and found that significant differences exist in the progression of joint destruction. Statistical analyses showed that joint destruction in the feet starts very early, being present even at the clinical onset of RA, but progresses slowly during the course of the disease. In contrast, joint destruction in the wrist starts slowly but progresses faster than that in the feet. Several factors were significantly associated with joint destruction evaluated by Larsen grade and SHS, but anti-CCP status was specifically associated with joint destruction in the wrist rather than that in the feet, while RF status was not associated with joint destruction in either the wrist or the feet.

The suppression of the progression of joint destruction has been the key target of therapeutic strategies in RA treatment. Joint destruction has been universally evaluated using SHS as the sum of joint destruction in the hands/wrists and the feet, but the differences between the two anatomical regions have not been sufficiently distinguished. Bakker *et al*. previously reported that the longitudinal relationship between DAS28 score and radiographic progression was influenced by the region of progression, and that radiographic progression in predominantly foot progressors was significantly greater than that of predominantly hand progressors or hand/foot similar progressors [25]. Another report also shows that destruction of the MTP joint is worsened and related to the walking ability despite of the decrease of disease activity and symptoms up to eight years after the onset of the disease [26]. Our current results support these observations, and further, these provide evidence of more detailed subgroups associated with joint destruction of the wrists and the feet, plus individual risk factors for the two sites of joint destruction.

Larsen grade is an established evaluation method for joint destruction in patients with RA, although the total SHS score and its modifications have been used for most clinical studies. SHS is one of the most reliable methods for analysis of joint destruction in the wrist/hand and the feet because of its comprehensiveness and its precision in evaluating the whole picture of joint destruction. However, the total SHS score and its modifications have undeniable limitations for analysis of individual joints in the wrists and the hands, and do not grade the joints according to the degree of destruction. Therefore, we decided to use the Larsen grade to evaluate the entire wrist joint and the MTP joints, and then used the results of the SHS to evaluate those obtained by use of the Larsen grade. The results obtained with the Larsen grade and those obtained with the SHS showed some variation in the differences between joint destruction in the wrist and the feet (Fig 2B and S1 Fig) and in the indicators for joint destruction (Table 3 and S1 Table), but we found close similarities in the results evaluated by the two methods, indicating that the results shown here are sufficiently reliable.

There have been many indicators reported previously to predict joint destruction, including sex (female)[15], IL-6 [2], MMP-3 [27, 28], bone density and its loss [29, 30], bone and cartilage biomarkers [28, 31, 32] and the first year progression [2]. We recently reported a main contribution of DRB1*04:05 among shared epitope and involvement of 57^th^ DRB1 amino acid in association with joint destruction [33]. Among these, both anti-CCP and RA status are the most frequently discussed and analysed predictors of both joint destruction and/or remission [2, 14–17, 34]. However, the differences in these two well-known serological markers have not been sufficiently evaluated, especially in terms of the regional effects of joint destruction. This study demonstrates, for the first time, that anti-CCP status is a strong indicator of joint destruction in the wrist, and is especially an indicator of more severe destruction of the wrist than of the feet. In contrast, in this study RF status was not a predictor of joint destruction in either the wrist or the feet. The reasons for the contradictory results of this study compared with previous reports regarding RF status may be that we analysed the wrist (the wrist/hands) and the feet separately, which lessened the effects of the sero-positivity compared with cases when both anatomical regions were analysed in combination. This observation also indicates that anti-CCP status should be considered a stronger indicator of joint destruction than RF status. Further studies are needed to obtain more precise predictability using anti-CCP levels or other new biomarkers.

This study has several limitations. First, because the study was cross-sectional, the progression of joint destruction may be dependent on other confounders, particularly changes in treatment strategies and medication, although in this study medication status did not affect joint destruction. Second, we specifically focused on joint destruction of the wrist and the MTP joints: destruction of other joints may progress differently. Third, it has not been scientifically demonstrated that the cut-off point for joint destruction that we decided to use, which included one-third of the cohort, is appropriate for analysis of more severe destruction. Predictors for milder or more severe joint destruction should be analysed in further studies. Lastly, the regional differences in joint destruction should be considered from the perspective of disability in daily life. It is necessary to obtain longitudinal results for the effects of each joint on disability, and then to establish therapeutic strategies by utilizing medication and other therapeutic modalities for individual joints. We expect further studies would clarify these points.

In conclusion, joint destruction in the feet started earlier than that in the wrists in patients with RA, while the latter progresses faster over the duration of the disease. Anti-CCP status is a strong indicator of joint destruction in the wrists, while RF status is not. Anti-CCP sero-positivity predicts joint destruction in the wrists better than in the feet.

## Supporting Information

S1 Fig Comparison of difference of the joint destruction between the wrist and the feet in SHS. *P* < 0.001. *SHS*: the modified Sharp/van der Heijde score.
(TIFF)

S1 Table The number and the percentages of each Larsen grade in each quadrant subgroup divided by the duration of the disease. (DOCX)

S2 Table Prognostic factors of the joint destruction in SHS and covariables (OR, 95%CI). *SHS;* the modified Sharp/van der Heijde method, *OR;* odds ratio, *95%CI;* 95% confidence interval, *DAS28;* Disease Activity Score 28, *anti-CCP;* anti-cyclic citrulinated peptide antibody, *RF;* rheumatoid factor, *: p<0.05.
(DOCX)
