# Supplementary figures and images for: Serological and Progression Differences of Joint Destruction in the Wrist and the Feet in Rheumatoid Arthritis - A Cross-Sectional Cohort Study
Source: PLoS One. 2015 Aug 28;10(8):e0136611. doi: 10.1371/journal.pone.0136611 (PMC4552680; doi:10.1371/journal.pone.0136611)

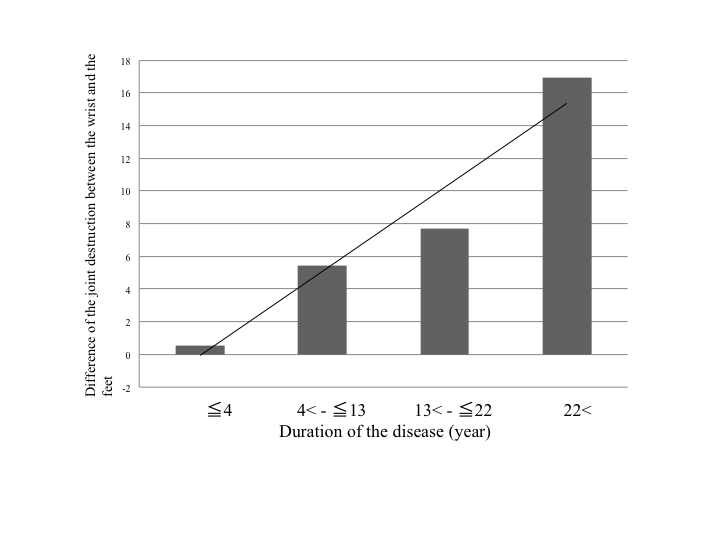

Supplement: S1 Fig — P < 0.001. SHS: the modified Sharp/van der Heijde score. (TIFF) [file pone.0136611.s001.tiff]
