# Supplementary material for: Serological and Progression Differences of Joint Destruction in the Wrist and the Feet in Rheumatoid Arthritis - A Cross-Sectional Cohort Study
Source: PLoS One. 2015 Aug 28;10(8):e0136611. doi: 10.1371/journal.pone.0136611 (PMC4552680; doi:10.1371/journal.pone.0136611)
Supplement: S1 Table — (DOCX) [file pone.0136611.s002.docx]

|  | ≦4 (n=93) | | 4<-≦13 (n=87) | | 13<-≦22 (n=74) | | 22< (n=60) | |
| --- | --- | --- | --- | --- | --- | --- | --- | --- |
|  | toe | wrist | toe | wrist | toe | wrist | toe | wrist |
| Larsen 0-1 | 26  (28%) | 78  (83.9%) | 26  (29.9)% | 39  (44.8%) | 26  (35.1%) | 23  (31.1%) | 15  (25%) | 9  (15%) |
| Larsen 2 | 51  (54.8%) | 7  (7.5%) | 36  (41.3%) | 23  (26.4%) | 18  (24.3%) | 15  (20.2%) | 16  (26.7%) | 3  (5%) |
| Larsen 3 | 7  (7.5%) | 3  (3.2%) | 13  (14.9%) | 15  (17.2%) | 12  (16.2%) | 9  (12.2%) | 16  (26.7%) | 4  (6.7%) |
| Larsen 4 | 3  (3.2%) | 2  (2.2%) | 8  (9.2%) | 7  (8%) | 4  (5.4%) | 16  (21.6%) | 5  (8.3%) | 22  (36.7%) |
| Larsen 5 | 6  (6.4%) | 3  3.2%) | 4  (4.3%) | 3  (3.4%) | 14  (18.9%) | 11  (14.9%) | 8  (13.3%) | 22  (36.7%) |

S1 Table The number and the percentages of each Larsen grade in each quadrant subgroup divided by the duration of the disease
