# Supplementary material for: Serological and Progression Differences of Joint Destruction in the Wrist and the Feet in Rheumatoid Arthritis - A Cross-Sectional Cohort Study
Source: PLoS One. 2015 Aug 28;10(8):e0136611. doi: 10.1371/journal.pone.0136611 (PMC4552680; doi:10.1371/journal.pone.0136611)
Supplement: S2 Table — SHS; the modified Sharp/van der Heijde method, OR; odds ratio, 95%CI; 95% confidence interval, DAS28; Disease Activity Score 28, anti-CCP; anti-cyclic citrulinated peptide antibody, RF; rheumatoid factor, *: p<0.05. (DOCX) [file pone.0136611.s003.docx]

| S2 Table Prognostic factors of the joint destruction in SHS and covariables (OR, 95%CI) | | |
| --- | --- | --- |
|  | Wrists/hands ≧56 | Feet ≧16.6 |
| Duration of the disease | 1.14 (1.11-1.19)* | 1.10 (1.07-1.14)* |
| Anti-CCP positivity | 4.48 (1.83-11.97)* | 2.41 (1.12-5.44)* |
| RF positivity | 1.48 (0.65-3.50) | 1.69 (0.80-3.69) |

*SHS;* the modified Sharp/van der Heijde method*, OR;* odds ratio, *95%CI;* 95% confidence interval, *DAS28;* Disease Activity Score 28, *anti-CCP;* anti-cyclic citrulinated peptide antibody, *RF;* rheumatoid factor, *: p<0.05
